# MicroRNA-424-5p inhibits the proliferation, migration, and invasion of nasopharyngeal carcinoma cells by decreasing AKT3 expression

**DOI:** 10.1590/1414-431X20209029

**Published:** 2020-06-03

**Authors:** Chong Zhao, Feng Zhao, Huiying Chen, Yuehua Liu, Jiping Su

**Affiliations:** 1 Guangxi Medical University, Nanning, China; 2 Department of Otorhinolaryngology and Head and Neck Surgery, Affiliated Hospital of Southwest Medical University, Luzhou, China; 3 Department of Otorhinolaryngology and Head and Neck Surgery, First Affiliated Hospital of Guangxi Medical University, Nanning, China

**Keywords:** Nasopharyngeal carcinoma, microRNA-424-5p, AKT3

## Abstract

This study examined the expression and potential mechanism of microRNA (miRNA)-424-5p in nasopharyngeal carcinoma (NPC). NPC tissues were collected from 40 patients who were enrolled in the study, and skin samples were collected from 26 healthy subjects during plastic surgery as controls. We performed various *in vitro* assays using miR-424-5p to examine its function in primary NPC-1 cells. Bioinformatics was employed to analyze potential target genes and signaling pathways of miR-424-5p. We found that miR-424-5p expression in NPC tissues is downregulated and negatively correlated with lymph node metastasis and clinical staging. Expression of miR-424-5p in NPC cells was also downregulated, and transfection with miR-424-5p mimics inhibited proliferation, migration, and invasion of NPC-1 cells. Bioinformatics identified the AKT3 gene as a potential target of miR-424-5p and dual luciferase assays confirmed this finding. Upregulation of AKT3 expression rescued the inhibitory effect of miR-424-5p on the proliferation, migration, and invasion. Our results suggest that miR-424-5p inhibited the proliferation, migration, and invasion of NPC cells by decreasing AKT3 expression.

## Introduction

Nasopharyngeal carcinoma (NPC) is a type of head and neck cancer with a high incidence in southern China and Southeast Asia (1). Similar to most tumors, the occurrence of NPC is closely related to genetic factors, infection by virus such as Epstein-Barr virus, and environmental factors (2). Low-differentiation squamous cell carcinoma is the common clinical type of NPC, while highly differentiated squamous cell carcinoma, adenocarcinoma, and alveolar cell carcinoma are rare types of NPC (3). Because of the special anatomical position of NPC, radiation therapy is often used by clinicians as the first choice for treatment (4). In recent years, the progress of radiotherapy and chemotherapy and the development of new tumor therapies such as immunotherapy and precision medicine have greatly improved the treatment options for NPC. However, a considerable number of NPC patients still show drug resistance, recurrence, and tumor metastasis, which seriously restrict the effects of clinical treatment for the disease (5,6). Studies have shown that the recurrence and metastasis of NPC are the main causes of death (7). Invasion and metastasis of NPC is a multi-gene, multi-stage, and multi-step complex process that involves many factors (7). In addition, the molecular mechanism of NPC remains to be completely elucidated (8). Therefore, clarifying the molecular mechanism of invasion and metastasis of NPC to search for molecular targets that are related to the malignant biological behaviors of NPC is critical for improving the clinical treatment of NPC.

MicroRNA (miRNA) is a class of non-encoding small RNA molecules that are 18-25 nucleotides in length. miRNAs interact with the 3′-untranslated region (UTR) of target mRNAs to form transcriptional silencing complexes, organize the translation of proteins, and regulate the expression of genes (9,10). Several studies have demonstrated various roles for miRNAs in cancer, and miRNAs can function either as oncogenes or tumor suppressor genes to regulate the occurrence and development of tumors (11). Furthermore, aberrant expression of these oncogenic and tumor suppressive miRNA molecules has been identified in multiple cancers, leading to changes in cell proliferation, invasion, and metastasis (14,15). For example, miR-16 inhibits the proliferation and metastasis of hepatocellular carcinoma (12), and miR-25 promotes the proliferation and invasion of gastric cancer by regulating ERBB2 (13). Therefore, examining miRNA molecules that are abnormally expressed in tumors and participate in the invasion and metastasis of the tumors is of great clinical importance.

The has-miR-424-5p gene is a newly discovered miRNA located on the human Xq26.3 chromosome. miR-424-5p targets different genes in different tissues, suggesting its diversified functions (16). Abnormal expression of miR-424-5p has been detected in many tumor tissues. For example, miR-424-5p is upregulated in esophageal squamous cell carcinoma, colorectal cancer, and non-small cell lung cancer, but its expression in cervical cancer is downregulated, suggesting that miR-424-5p is differentially expressed in different tumors (17,18). Currently, the expression and function of miR-424-5p in NPC are still unclear. In the present study, we evaluated the expression and potential function of miR-424-5p in NPC.

## Material and Methods

### Patients

Forty NPC patients who received treatments at our hospital between January 2015 and January 2016 were enrolled in the present study. The patient group included 21 males and 19 females (mean, 48.5 years; range 31-68 years). None of the patients had a history of other types of malignant tumors, chemotherapy, or radiotherapy. Among all patients, 27 had cervical lymph node metastasis and 13 had no cervical lymph node metastasis. Based on TNM staging and grading standards by the Union for International Cancer Control in 2003, 11 cases were at stage I, 9 were at stage II, 15 were at stage III, and 5 were at stage IV. According to pathological classification, 35 cases had poorly differentiated squamous cell carcinoma and 5 had highly differentiated squamous cell carcinoma. NPC tissues were collected from the 40 patients as the experimental group and skin samples collected from 26 healthy subjects during plastic surgery were used as controls. All procedures were approved by the Ethics Committee of Guangxi Medical University. Written informed consent was obtained from all patients or their families.

### Primary cell culture

Excised human NPC tissues were washed with phosphate-buffered saline and soaked in penicillin (5×10^5^ U/L) and streptomycin (100 mg/L) for 20 min. The tissues were cut into 1-mm^3^ pieces and digested with 30 mL collagenase I (2×10^5^ U/L) at 37°C for 2 h. After filtration with a 100-mesh cell sieve, the filtrate was centrifuged at 1000 *g* for 10 min at room temperature before discarding the supernatant. The pellet was washed twice with phosphate-buffered saline and resuspended with RPMI-1640 medium supplemented with 10% fetal bovine serum in culture flasks with a bottom area of 25 cm^2^. Immortalized nasopharyngeal epithelial NP69 cells (Cell Bank, Chinese Academy of Sciences, China) were cultured in RPMI-1640 medium supplemented with 10% fetal bovine serum, 100 IU/mL penicillin, and 100 IU/mL streptomycin at 37°C, 5% CO_2_, and 70% humidity. The cells were passaged every 3 days.

### Transfection of miRNA mimics

One day before transfection, NPC-1 cells (2×10^5^) in logarithmic growth were seeded into 24-well plates containing antibiotic-free RPMI-1640 medium with 10% fetal bovine serum for culture until cells reached 70% confluency. In one vial, 1.5 µL miR-negative control (20 pmol/µL; miR-NC group) or miR-424-5p mimics (20 pmol/µL; miR-424-5p mimics group) (Hanbio Biotechnology Co., Ltd., China) were mixed with 50 µL Opti Mem medium (Thermo Fisher Scientific, USA). In a second vial, 1 µL Lipofectamine 2000 (Thermo Fisher Scientific) was mixed with 50 µL Opti Mem medium. After being allowed to stand for 5 min, the two vials were combined and held at room temperature for 20 min. The mixtures were added onto the appropriate cells. Six hours later, the medium was replaced with RPMI-1640 medium containing 10% fetal bovine serum. After culture for 48 h, the cells were collected for further assays.

### Quantitative real-time polymerase chain reaction (qRT-PCR)

NPC tissues (100 mg) were ground into powder in liquid nitrogen and lysed using 1 mL TRIzol reagent (Thermo Fisher Scientific) following the manufacturer's manual. Total RNA was extracted using phenol chloroform. The concentration and quality of RNA was measured using ultraviolet spectrophotometry (Nanodrop ND2000, Thermo Scientific). cDNA was obtained by reverse transcription from 1 μg RNA and samples were stored at −20°C. Reverse transcription of miRNA was carried out using the miScript II RT Kit (Qiagen, Germany) following the manufacturer's manual.

The expression of miR-424-5p was determined with the miScript SYBR Green PCR Kit (Qiagen), using U6 as an internal reference. The forward primer sequence of miR-424-5p primer was 5′-CAGCAGCAATTCATGTTTTGAA-3′ and its reverse primer sequence was universal and not provided by the supplier. The sequences of the U6 primers were forward, 5′-CTCGCTTCGGCAGCACA-3′ and reverse 5′-AACGCTTCACGAATTTGCGT-3′. The reaction system (20 μL) contained 10 μL qRT-PCR-Mix, 0.5 μL forward primer, 0.5 μL reverse primer, 2 μL cDNA, and 7 μL ddH_2_O. The reaction protocol was as follows: initial denaturation at 95°C for 10 min, followed by 40 cycles of denaturation at 95°C for 1 min, and annealing at 60°C for 30 s (iQ5; Bio-Rad, USA). The 2^−ΔΔCt^ method (19) was used to calculate the relative expression of miR-424-5p against U6. Each sample was tested in triplicate.

### Nude mouse model of subcutaneous tumorigenesis

Five nude mice were selected randomly and received subcutaneous inoculation with primary NPC cells (1×10^6^). The mice in the control group were injected with an equal amount of saline. The next day, the injection sites in nude mice were observed to check the degree of absorption. The tumor size was observed and measured every day.

### Construction of nude mouse model of lung metastasis

Eight male nude mice were randomly divided into two groups, and NPC-1 (5×10^5^) and NPC-2 (5×10^5^) cells were injected into each nude mouse via tail vein. Nude mice were observed daily and executed 14 days later. The lung tissue was dissected, and the lung cancer nodules were observed. The tissues were stained with hematoxylin and eosin.

### CCK-8 assay

NPC-1 cells were seeded at a density of 2000/well in 96-well plates. At 0, 24, 48, and 72 h later, 20 μL CCK-8 reagent (5 g/L; Beyotime, China) was added to the cells. After incubation at 37°C for 2 h, the absorbance of each well was measured at 490 nm and cell proliferation curves were plotted. Each group was tested in three replicate wells and the values were averaged.

### Transwell assay

Cell migration and invasion were determined using 24-well transwell plates (Corning Inc., USA) with 8-μm-pore polycarbonate membranes. Matrigel (BD Biosciences, USA) was thawed at 4°C overnight and diluted with serum-free RPMI-1640 medium (dilution 1:2). The mixture (50 μL) was evenly smeared into the upper chamber (Merck Millipore, USA) and the chambers were incubated at 37°C for 1 h. After solidification, 1×10^5^ cells from each group were seeded into the upper chamber containing 200 μL serum-free RPMI-1640 medium. Next, 500 μL RPMI-1640 medium supplemented with 10% fetal bovine serum was added into the lower chamber. After 24 h, the chamber was removed and the cells in the upper chamber were wiped off. After fixation with 4% formaldehyde for 10 min, the membrane was stained using the Giemsa method for microscopic observation. The number of migrated cells was calculated and averaged in five random fields (200×). All procedures were carried out on ice with pipetting tips precooled at 4°C.

### Bioinformatics

We searched http://www.targetscan.orgwebsite for “miR-424-5p” to identify potential target genes. These potential target genes were submitted to the David database (https://david.ncifcrf.gov/), and the keyword “human” was selected. The potential downstream signaling pathways were analyzed.

### Western blotting

NPC-1 cells were trypsinized and collected. Cells (1×10^6^) in each group were lysed with precooled radio-immunoprecipitation assay (RIPA) lysis buffer (600 μL; 50 mM Tris-base, 1 mM EDTA, 150 mM NaCl, 0.1% sodium dodecyl sulfate, 1% TritonX-100, 1% sodium deoxycholate; Beyotime Institute of Biotechnology, China) for 30 min on ice. The sample was centrifuged at 12,000 *g* and 4°C for 10 min. Protein concentration of the supernatant was determined by the bicinchoninic acid (BCA) protein concentration determination kit (RTP7102, Real-Times (Beijing) Biotechnology Co., Ltd., China). The samples were then mixed with 5× sodium dodecyl sulfate loading buffer before denaturation in a boiling water bath for 10 min. Samples (10 μL) were then subjected to separation by 10% sodium dodecyl sulfate-polyacrylamide gel electrophoresis at 100 V. The resolved proteins were transferred to polyvinylidene difluoride membranes on ice (250 mA, 1 h) and blocked with 50 g/L skimmed milk at room temperature for 1 h. Membranes were incubated with rabbit anti-human AKT3 polyclonal primary antibody (1:1000; ab152157; Abcam, UK) or mouse anti-human GAPDH monoclonal primary antibody (1:4000; Beyotime) at 4°C overnight. After extensive washing with phosphate-buffered saline with Tween 20 five times for 5 min each, the membranes were incubated with goat anti-rabbit or goat anti-mouse horseradish peroxidase-conjugated secondary antibodies (1:4,000; Abcam) for 1 h at room temperature before washing with phosphate-buffered saline with Tween 20 five times for 5 min each. The membrane was developed with an enhanced chemiluminescence detection kit (Beyotime) for imaging. Image Lab v3.0 software (Bio-Rad, USA) was used to acquire and analyze imaging signals. The relative content of the target protein was normalized to GAPDH.

### Dual luciferase reporter assay

Based on the bioinformatics results, wild-type (WT) and mutant seed regions of miR-424-5p in the 3′-UTR of AKT3 gene were chemically synthesized *in vitro*. *Spe-1* and *HindIII* restriction sites were included on either end and the fragment was then cloned into pMIR-REPORT luciferase reporter plasmids. Plasmids (0.5 μg) with WT or mutant 3′-UTR sequences were co-transfected with agomiR-negative control (NC) or agomiR-424-5p (100 nM; Sangon Biotech, China) into 293T cells. After cultivation for 24 h, the cells were lysed and processed using the dual luciferase reporter assay kit (Promega, USA) according to the manufacturer's manual. Fluorescence intensity was measured using GloMax 20/20 luminometer (Promega). The fluorescence values of each group of cells were measured using renilla fluorescence activity as an internal reference.

### Statistical analysis

The results were analyzed using SPSS 20.0 statistical software (IBM, USA). Data are reported as means±SD. Data were tested for normality. Multigroup measurement data were analyzed using one-way ANOVA. In case of homogeneity of variance, the least significant difference and Student-Newman-Keuls methods were used; in case of heterogeneity of variance, Tamhane's T2 or Dunnett's T3 method was used. Comparison between two groups was carried out using Student's *t*-test. P<0.05 indicated statistically significant differences.

## Results

### Expression of miR-424-5p was reduced in NPC tissues and correlated with the occurrence and development of NPC

**Figure 1:**
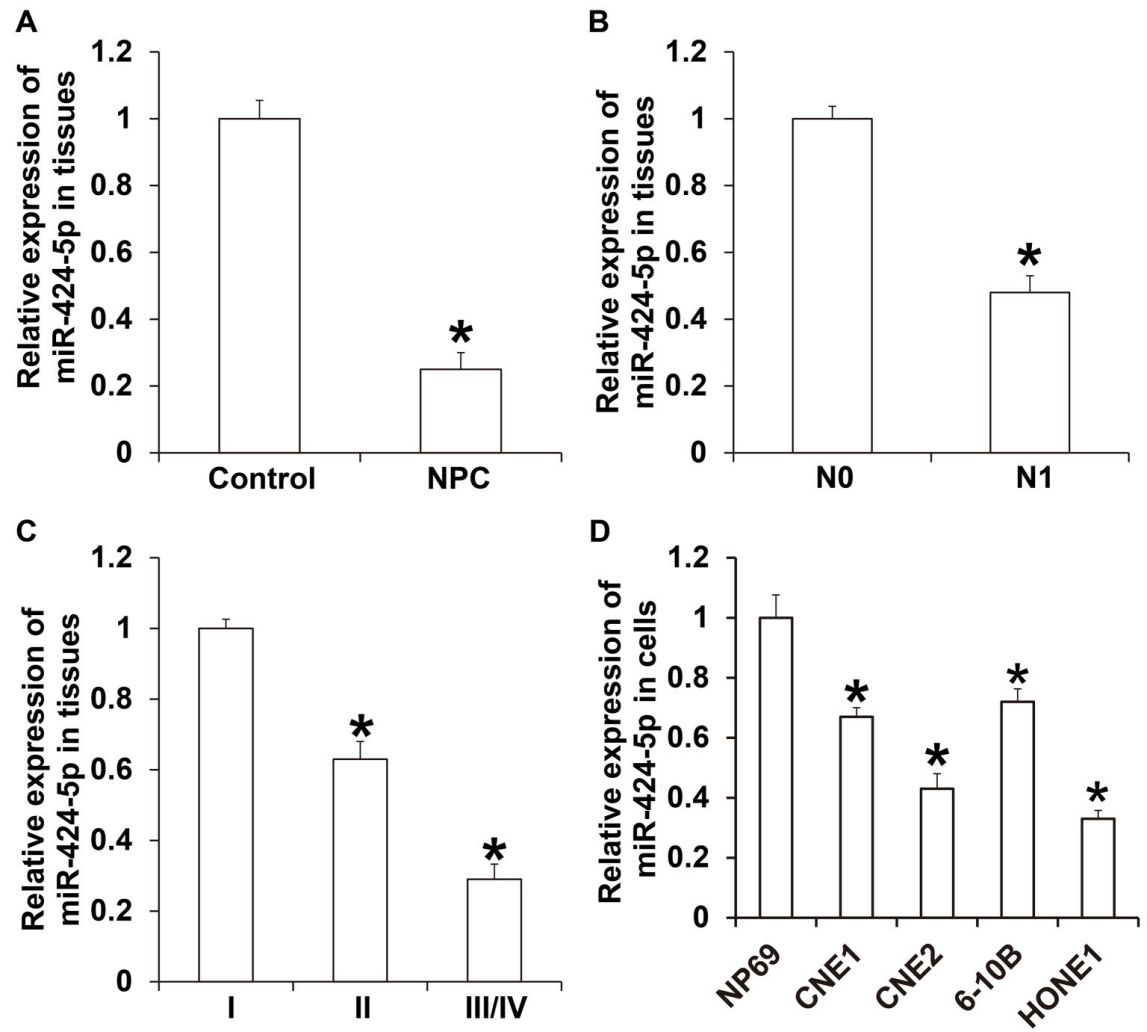
**A**, Expression of miR-424-5p in nasopharyngeal carcinoma (NPC) tissues and control tissues. *P<0.05 compared with control group. **B**, Expression of miR-424-5p in NPC tissues from patients with (N1) or without (N0) lymph node metastasis. *P<0.05 compared with the N0 group. **C**, Expression of miR-424-5p in NPC tissues from patients at TNM stages I, II, or III/IV. *P<0.05 compared with stage I. **D**, Expression of miR-424-5p in NPC cells and normal nasopharyngeal epithelial NP69 cell lines. Data are reported as means±SD. *P<0.05 compared with NP69 cells (*t*-test or ANOVA).

The relative expression of miR-424-5p in NPC tissues (0.25±0.05) was significantly lower than that in the control group (P<0.05) (Figure 1A). In addition, the relative expression of miR-424-5p in NPC tissues from patients with lymph node metastasis (0.48±0.05; N1 group) was significantly lower than that from patients without lymph node metastasis (N0 group; P<0.05) (Figure 1B). The relative expression of miR-424-5p in NPC tissues at TNM stage II or at stages III and IV was significantly lower than that in NPC tissues at stage I (P<0.05) (Figure 1C). We also observed that miR-424-5p expression in NPC cell lines was significantly lower than that in normal nasopharyngeal epithelial NP69 cells (P<0.05) (Figure 1D). These results suggested that the expression of miR-424-5p was reduced in NPC tissues and correlated with the occurrence and development of NPC.

### Isolation of primary NPC-1 and NPC-2 cells

**Figure 2:**
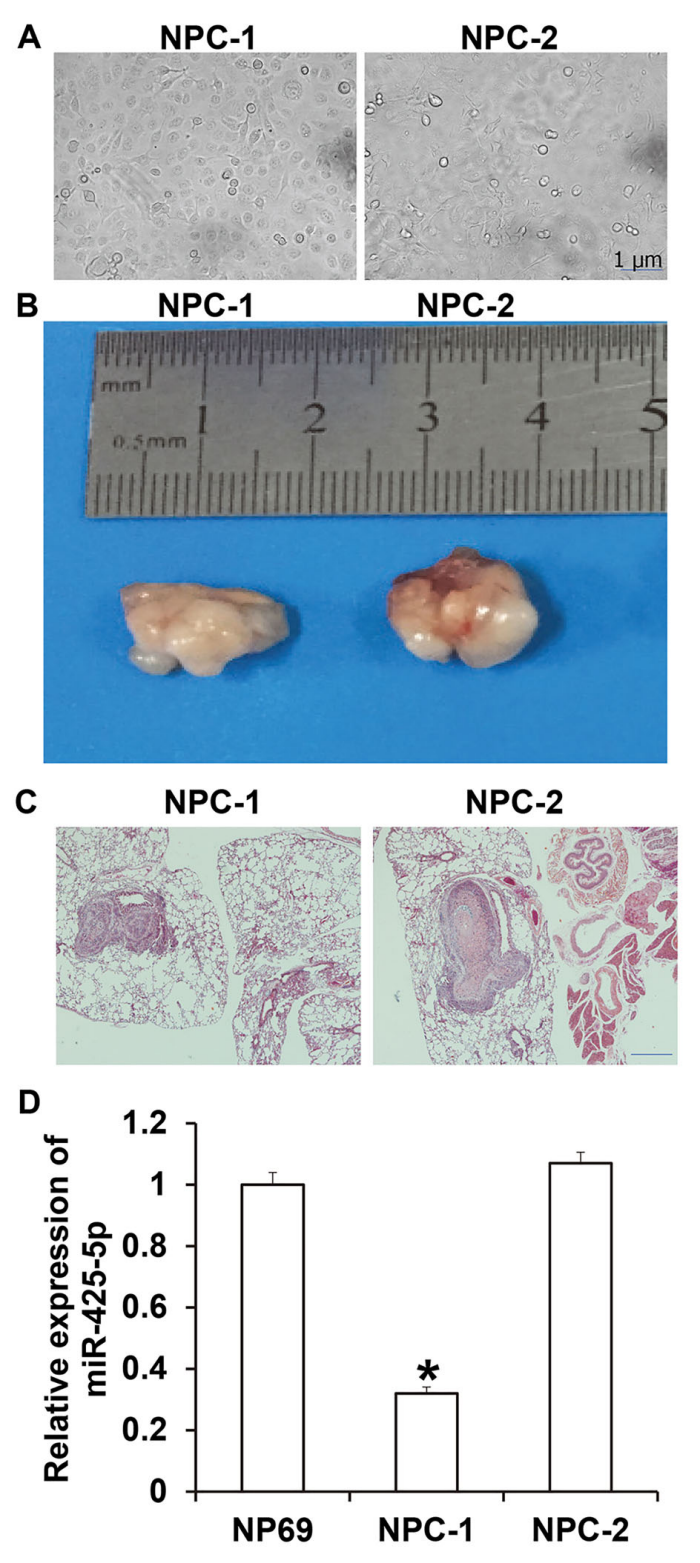
Identification of primary nasopharyngeal carcinoma (NPC) cells. **A**, Light microscopy of primary NPC-1 and NPC-2 cells (scale bar: 1 μm). **B**, Tumorigenicity of NPC-1 and NPC-2 in nude mice. **C**, Lung metastasis of NPC-1 and NPC-2 cells (scale bar: 100 μm). **D**, Expression of miR-425-5p in NPC-1 and NPC-2 cells. Data are reported as means±SD. *P<0.05 compared with NP69 cells (ANOVA).

The morphologies of NPC-1 and NPC-2 cells are shown in Figure 2A. We used these cell lines for subcutaneous inoculation in mice and observed tumors at injection sites on day 7 (Figure 2B). Lung metastasis tests showed that the inoculated NPC-1 and NPC-2 cells metastasized to the lungs (Figure 2C). qRT-PCR showed that the expression of miR-425-5p in NPC-1 cells was significantly lower than that in NP69 cells (P<0.05) (Figure 2D). The results suggested that primary NPC-1 and NPC-2 cells were successfully isolated.

### Overexpression of miR-424-5p inhibited proliferation of NPC-1 cells *in vitro*

**Figure 3:**
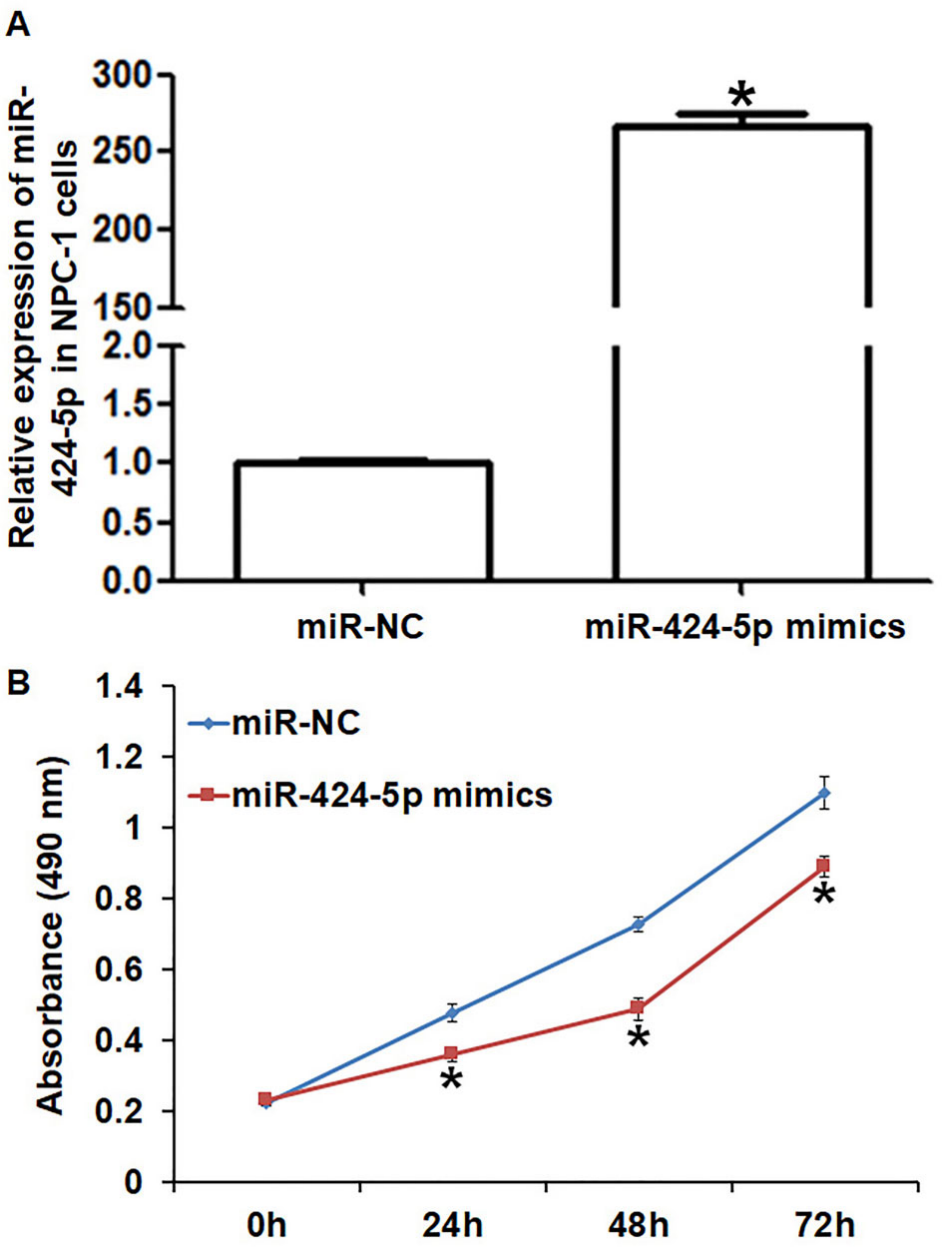
Effect of miR-424-5p on the proliferation of nasopharyngeal carcinoma (NPC)-1 cells. **A**, Expression of miR-424-5p in NPC-1 cells after transfection with miR-NC (negative control) or miR-424-5p mimics. **B**, Proliferation of NPC-1 cells after transfection with miR-NC or miR-424-5p mimics. CCK-8 assay was used to detect cell proliferation. Data are reported as means±SD. *P<0.05 compared with the miR-NC group (*t*-test).

miR-424-5p expression in the miR-424-5p mimics group was significantly higher than that in the miR-NC group (P<0.05) (Figure 3A). CCK-8 assay showed the miR-424-5p mimics group had significantly decreased growth at 24, 48, and 72 h compared with the miR-NC group (P<0.05 at all points) (Figure 3B). These results indicated that overexpression of miR-424-5p inhibited the proliferation of NPC-1 cells *in vitro*.

### Overexpression of miR-424-5p inhibited migration and invasion of NPC-1 cells *in vitro*

**Figure 4:**
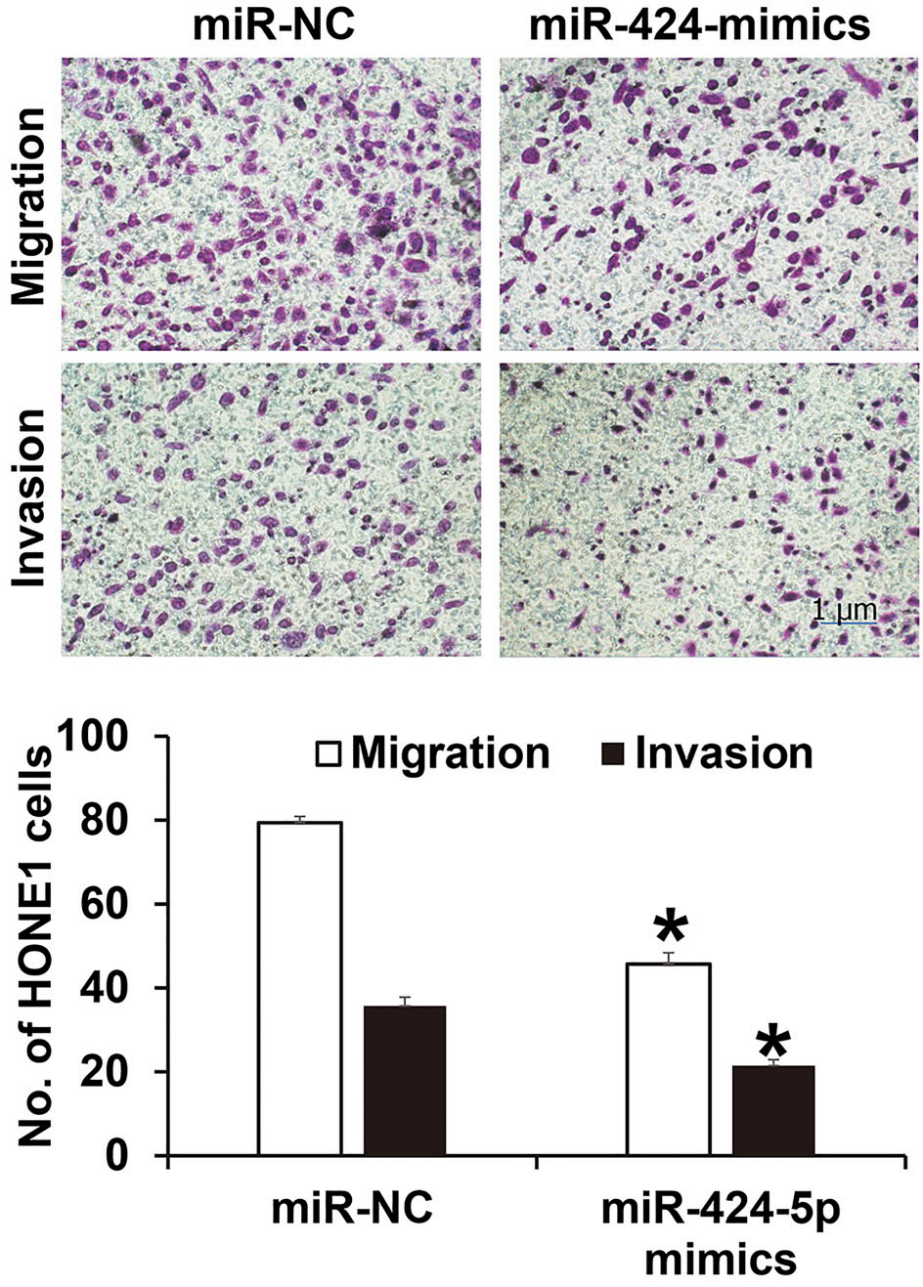
Effect of miR-424-5p on the migration and invasion of nasopharyngeal carcinoma (NPC)-1 cells. Transwell assay was used to determine migration and invasion (scale bar: 1 μm). Data are reported as means±SD. *P<0.05 compared with the miR-NC (negative control) group (*t*-test).

The number of NPC-1 cells that crossed the membrane in the miR-424-5p mimics group (45.7±2.7) was significantly lower than that in the miR-NC group (79.4±1.5) (P<0.05) (Figure 4). Invasion assay similarly showed that the number of NPC-1 cells that crossed the membrane in the miR-424-5p mimics group (21.5±1.4) was also significantly lower than that in the miR-NC group (35.7±2.1) (P<0.05). These results suggested that overexpression of miR-424-5p inhibited the migration and invasion of NPC-1 cells *in vitro*.

### miR-424-5p bound the 3′-UTR seed region of AKT3 mRNA to regulate its expression

The results identified 1507 potential target genes of miR-424-5p that were involved in MAPK, ErBb, and Ras signaling pathways (data not shown). Among all the potential target genes of miR-424-5p, AKT3 participated in the regulation of proliferation, apoptosis, and migration of cells and was involved in signaling pathways that were closely related to the occurrence and development of tumors, such as MAPK and ErRb signaling pathways (data not shown).

**Figure 5:**
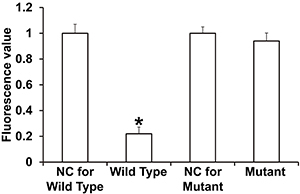
Identification of the interaction between miR-424-5p and AKT3 mRNA using dual luciferase reporter assay. Dual luciferase assay was performed in 293T cells transfected with reporter vectors with wild type or mutant 3′-UTR sequences along with agomiR-negative control (NC) or agomiR-424-5p. The fluorescence values of each group of cells were measured using renilla fluorescence activity as an internal reference. Data are reported as means±SD. *P<0.05 compared with the NC group (ANOVA).

To determine whether miR-424-5p targets the 3′-UTR of AKT3 mRNA, dual luciferase reporter assay was performed. The luciferase activity of cells co-transfected with agomiR-424-5p and the pMIR-REPORT-WT luciferase reporter plasmids, containing the wild-type 3′-UTR of AKT3 mRNA, was significantly lower than that in the negative control group (P<0.05). In contrast, the luciferase activity of cells co-transfected with agomiR-424-5p and the pMIR-REPORT-mutant luciferase reporter plasmid in which the binding sequence was mutated was not significantly different from that in the negative control group (P>0.05) (Figure 5). These results suggested that miR-424-5p bound the seed region in the 3′-UTR of AKT3 mRNA to regulate its expression.

### AKT3 rescued the inhibitory effect of miR-424-5p on the proliferation, migration, and invasion of NPC-1 cells

**Figure 6:**
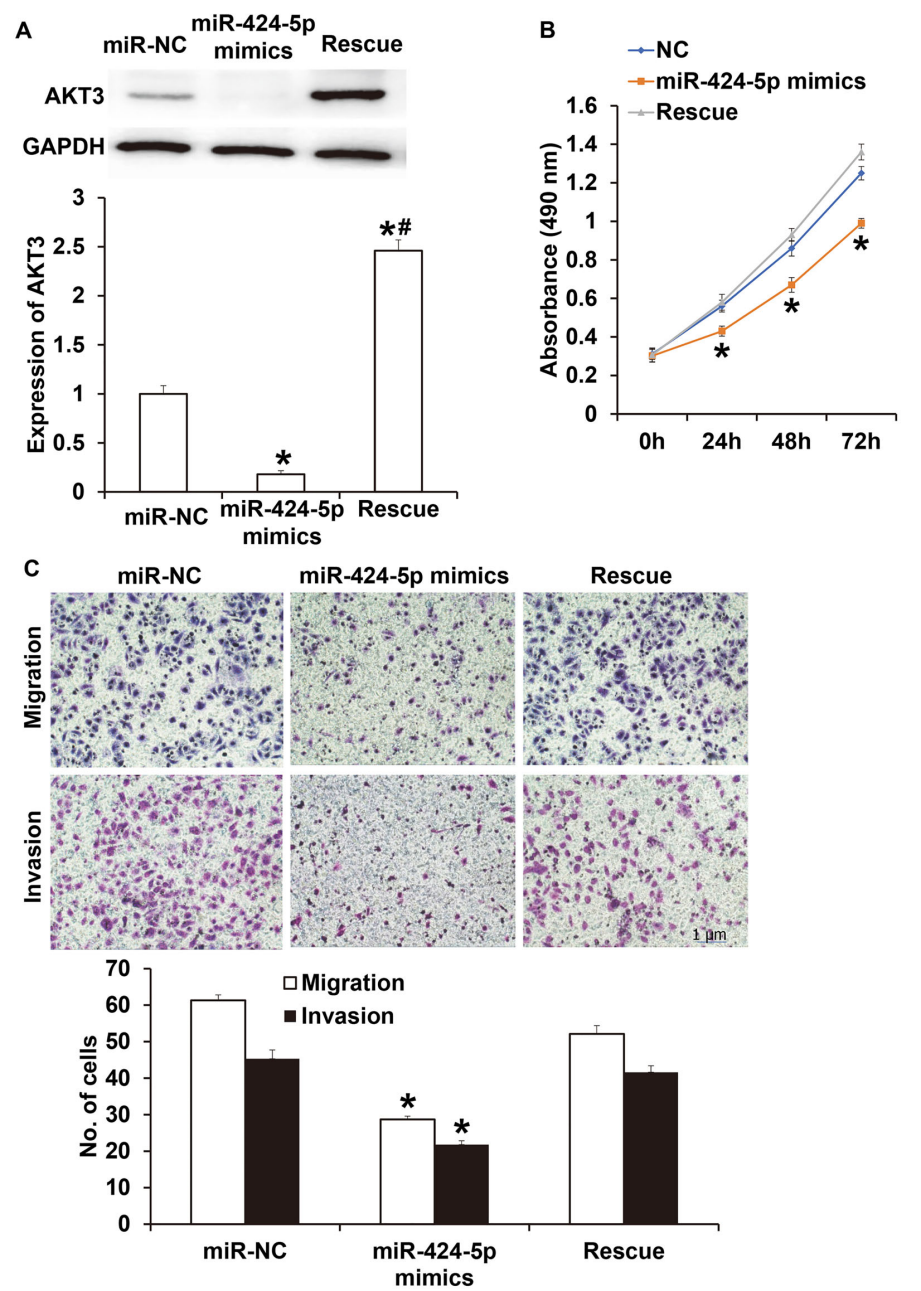
**A**, Expression of AKT3 cells in nasopharyngeal carcinoma (NPC)-1 cells transfected with miR-NC (negative control), miR-424-5p mimics, or miR-424-5p mimics+AKT3 (Rescue). Western blotting was used to determine AKT3 protein expression. **B**, Proliferation of NPC-1 cells transfected with miR-NC, miR-424-5p mimics, or miR-424-5p mimics+AKT3 (Rescue). CCK-8 assay was used to detect cell proliferation. **C**, Migration and invasion of NPC-1 cells transfected with miR-NC, miR-424-5p mimics, or miR-424-5p mimics+AKT3 (Rescue). Scale bar: 1 μm. Transwell assay was used to evaluate migration and invasion. Data are reported as means±SD. *P<0.05 compared with the miR-NC group; ^#^P<0.05 compared with the miR-424-5p mimics group (*t*-test).

AKT3 expression in NPC-1 cells transfected with miR-424-5p mimics was significantly lower than that in the miR-NC group (P<0.05) (Figure 6A). In addition, the level of AKT3 protein in NPC-1 cells transfected with both miR-424-5p mimics and AKT3 was significantly higher than that in miR-NC group or miR-424-5p mimics (P<0.05 for both). CCK-8 assay showed that the proliferation of NPC-1 cells transfected with miR-424-5p mimics was significantly reduced compared with that in the miR-NC group (P<0.05 at all points), while the proliferation of NPC-1 cells transfected with both miR-424-5p mimics and AKT3 was similar to that in the miR-NC group (P>0.05 at all points) (Figure 6B). Transwell assay showed that while the migration and invasion of NPC-1 cells transfected with miR-424-5p mimics were significantly lower than those in the miR-NC group (P<0.05 for both), the migration and invasion of NPC-1 cells transfected with both miR-424-5p mimics and AKT3 were not different from those in miR-NC group (P>0.05 for both) (Figure 6C). These results suggested that AKT3 rescued the inhibitory effect of miR-424-5p on the proliferation, migration, and invasion of NPC-1 cells.

## Discussion

NPC is the most common head and neck tumor in clinical settings and shows high malignancy. Cervical lymph node metastasis is prone to occur at the early stage of NPC. NPC tends to show resistance towards radiotherapy and chemotherapy, and the prognosis of NPC patients is poor (20). The recurrence and metastasis of NPC are the main contributing factors limiting its clinical treatment (21). Previous studies have shown that a large number of miRNA molecules are involved in the regulation of metastasis (22).

miR-424-5p is a newly discovered tumor-associated miRNA that is located at human Xq26.3. miR-424-5p is expressed by processing the 5′-terminus of has-miR-424 (16). Several studies have shown that the expression and biological functions of miR-424-5p vary across different tumors. For example, the expression of miR-424-5p is reduced in liver cancer, gastric cancer, and osteosarcoma tissues, and miR-424-5p inhibits the proliferation and metastasis of these tumor cells (23). In contrast, expression of miR-424-5p is increased in squamous cell carcinoma of the esophagus, breast cancer, and pancreatic cancer, and it promotes the occurrence and development of these tumors (24). In the present study, we showed that miR-424-5p expression was significantly reduced in NPC tissues and negatively correlated with lymph node metastasis and TNM staging. Moreover, we isolated primary NPC cells with low expression of miR-425-5p, particularly NPC-1 cells. *In vitro* functional analyses showed that upregulation of miR-424-5p expression inhibits the proliferation, migration, and invasion of NPC-1 cells, suggesting that miR-424-5p has oncogenic function and that reduced expression of miR-424-5p in NPC may be related to the occurrence and development of NPC. The target genes of miR-424-5p are distinct in different tumors, and this may be the main reason for different the biological functions of miR-424-5p. For example, Zhou et al. reported that miR-424-5p regulates the Notch signaling pathway by targeting KDM5B and has tumor suppressor functions in cervical cancer (25). Wei et al. (18) showed that miR-424-5p regulates the TGF-β signaling pathway by targeting Smad3 and promotes the proliferation of gastric cancer cells. Our bioinformatics results revealed the *AKT3* gene as a potential target of miR-424-5p. AKT is involved in the regulation of multiple downstream signaling pathways such as MAPK, Ras, and ErbB. Our qRT-PCR and western blotting data demonstrated that miR-424-5p downregulated the expression of AKT3 mRNA and protein. AKT is a key kinase in the PI3K/AKT signaling pathway and has three subtypes, AKT1, AKT2, and AKT3 (26). The expression of AKT3 is elevated in a variety of tumors and is associated with abnormal proliferation, apoptosis resistance, and poor prognosis of the tumors (27). For example, AKT3 can make 40-60% non-hereditary melanoma cells survive, and inhibition of AKT3 activity promotes the death of these cells (28). In addition, downregulation of AKT3 inhibits invasion and metastasis of glioma (29). The present study showed that elevation of AKT3 expression reversed the inhibitory effect of miR-424-5p on NPC cells and promoted the proliferation, migration, and invasion of NPC cells. Notably, the downstream signaling pathways of AKT3 and relevant molecules have not yet been identified. We plan on examining how AKT3 is related to the prognosis and clinical pathological characteristics of NPC and the correlation between AKT3 and miR-424-5p in clinical practice in future studies.

In conclusion, the present study demonstrated that miR-424-5p inhibited the proliferation, migration, and invasion of NPC cells by targeting AKT3 expression. The downregulation of miR-424-5p in NPC tissues was correlated with the occurrence and development of NPC.
